# Identification of Key Genes as Early Warning Signals of Acute Myocardial Infarction Based on Weighted Gene Correlation Network Analysis and Dynamic Network Biomarker Algorithm

**DOI:** 10.3389/fimmu.2022.879657

**Published:** 2022-06-20

**Authors:** Chenxi Song, Zheng Qiao, Luonan Chen, Jing Ge, Rui Zhang, Sheng Yuan, Xiaohui Bian, Chunyue Wang, Qianqian Liu, Lei Jia, Rui Fu, Kefei Dou

**Affiliations:** ^1^ Cardiometabolic Medicine Center, Department of Cardiology, Fuwai Hospital, National Center for Cardiovascular Diseases, Chinese Academy of Medical Sciences and Peking Union Medical College, Beijing, China; ^2^ State Key Laboratory of Cardiovascular Disease, Beijing, China; ^3^ Key Laboratory of Systems Biology, Shanghai Institute of Biochemistry and Cell Biology, Center for Excellence in Molecular Cell Science, Chinese Academy of Sciences, Shanghai, China; ^4^ Shanghai Immune Therapy Institute, Renji Hospital, Shanghai Jiao Tong University School of Medicine, Shanghai, China

**Keywords:** CAD, monocyte, transcriptomics, WGCNA, DNB

## Abstract

**Purpose:**

The specific mechanisms and biomarkersunderlying the progression of stable coronary artery disease (CAD) to acute myocardial infarction (AMI) remain unclear. The current study aims to explore novel gene biomarkers associated with CAD progression by analyzing the transcriptomic sequencing data of peripheral blood monocytes in different stages of CAD.

**Material and Methods:**

A total of 24 age- and sex- matched patients at different CAD stages who received coronary angiography were enrolled, which included 8 patients with normal coronary angiography, 8 patients with angiographic intermediate lesion, and 8 patients with AMI. The RNA from peripheral blood monocytes was extracted and transcriptome sequenced to analyze the gene expression and the differentially expressed genes (DEG). A Gene Oncology (GO) enrichment analysis was performed to analyze the biological function of genes. Weighted gene correlation network analysis (WGCNA) was performed to classify genes into several gene modules with similar expression profiles, and correlation analysis was carried out to explore the association of each gene module with a clinical trait. The dynamic network biomarker (DNB) algorithm was used to calculate the key genes that promote disease progression. Finally, the overlapping genes between different analytic methods were explored.

**Results:**

WGCNA analysis identified a total of nine gene modules, of which two modules have the highest positive association with CAD stages. GO enrichment analysis indicated that the biological function of genes in these two gene modules was closely related to inflammatory response, which included T-cell activation, cell response to inflammatory stimuli, lymphocyte activation, cytokine production, and the apoptotic signaling pathway. DNB analysis identified a total of 103 genes that may play key roles in the progression of atherosclerosis plaque. The overlapping genes between DEG/WGCAN and DNB analysis identified the following 13 genes that may play key roles in the progression of atherosclerosis disease: SGPP2, DAZAP2, INSIG1, CD82, OLR1, ARL6IP1, LIMS1, CCL5, CDK7, HBP1, PLAU, SELENOS, and DNAJB6.

**Conclusions:**

The current study identified a total of 13 genes that may play key roles in the progression of atherosclerotic plaque and provides new insights for early warning biomarkers and underlying mechanisms underlying the progression of CAD.

## Introduction

Coronary artery disease (CAD) remains the leading cause of disease burden worldwide (1). Based on clinical presentation, myocardium injury biomarkers, electrocardiography characteristics, and the extent of myocardium injury, CAD is generally classified as stable CAD and acute coronary syndrome (ACS), which included unstable angina and acute myocardial infarction (AMI). Stable CAD is primarily caused by lumen stenosis caused by atherosclerotic plaque and subsequent oxygen demand-supply mismatch, while ACS is the rupture of vulnerable plaque and subsequent occlusive thrombosis formation and myocardial necrosis (2).

However, the exact mechanism underlying the formation, progression, and rupture of plaque is unclear. The current well-established mechanism of this process involves lipid-driven inflammation (3). Monocyte-derived macrophages are one type of the key inflammatory cells within the plaque and participated in each stage of atherosclerosis plaque formation (4). The rapid development of high-throughput omics technology, such as genomics, transcriptomics, proteomics, and metabolomics, provides new insights into the mechanisms and biomarkers of CAD. For instance, peripheral RNA expression differed significantly between CAD patients and normal control (5) and within CAD patients (6).

There has been rich evidence in studies on biomarkers for diagnosis and risk stratification of coronary heart disease. The validated biomarkers are related to different pathophysiological processes of coronary heart disease, including myocardial injury, altered myocardial stress, inflammation, and vascular endothelial dysfunction. For example, cardiac troponin T (cTnT) and cardiac troponin I (cTnI) demonstrate myocardial tissue specificity (7) and are released into the blood when myocardial tissue suffers damage, leading to an elevated concentration level in peripheral blood (8). Likely, a heart-type fatty acid-binding protein is released into peripheral blood during AMI, and studies confirm its prognostic value for patients with suspected ACS and negative cardiac troponin test results (9, 10). The natriuretic peptide family is a set of typical biomarkers associated with myocardial stress. In 2017, B-type natriuretic peptide (BNP) and N-terminal pro-B-type natriuretic peptide (NT-proBNP) were recommended for the diagnosis, evaluation, and management of heart failure patients by AHA (11). Also, a recent study shows that BNP and NT-proBNP can guide risk stratification in patients with coronary heart disease (12). The soluble suppressor of tumorgenicity 2 (sST2) is a typical biomarker associated with inflammation and is proved to be an independent risk factor for long-term all-cause death in a stable coronary disease cohort (13). Endothelin, converted from its relatively stable primer big endothelin-1, has a strong constrictive effect on the coronary arteries, leading to endothelial dysfunction (14, 15). Research evidence shows that big endothelin–1 has the ability to predict long–term prognosis for both stable coronary heart disease and acute myocardial infarction (16, 17).

However, previous studies mainly focused on the biomarkers associated with the occurrence of CAD, while the biomarkers associated with the progression of CAD remain lacking.

Weighted correlation network analysis (WGCNA), proposed by scholars Zhang and Horvath, is an efficient and accurate bioinformatics method for analyzing microarray data (18). WGCNA analysis divides genes into several modules based on the similarity of gene expression profile and identifies the gene module and corresponding hub genes that are highly correlated with the clinical trait of interest.

WGCNA methods have been successfully applied to identify hub genes in many diseases, including cardiovascular disease (19), cancer (20), psychological disease (21), etc.

However, the primary objective of WGCNA, like most traditional bioinformatics methods, is to distinguish a disease state from a normal state or to diagnose the disease state rather than the “predisease” state (22). In other words, WGCNA may fail to accurately predict the early onset of disease before its development. To overcome this limitation, Chinese scholars Chen et al. proposed the dynamic network biomarker (DNB) theory based on the dynamic features of molecules within the biological system (23). Compared with traditional bioinformatics methods such as WGCNA, the DNB method mainly aims to diagnose the “predisease state,” which can detect the early warning signals and achieve early diagnosis before the onset of disease. The DNB method has been successfully used to detect the critical transition point and key molecules in many pathological process (22, 24–26). We hypothesize that the combined use of both WGCNA and the DNB method will contribute to the identification of robust biomarkers to predict disease deterioration.

The current study aims to explore novel gene biomarkers as early warning signals of AMI by analyzing the gene expression profile of peripheral blood monocytes in different stages of CAD based on transcriptomic sequencing technology, combined with both WGCNA and DNB methods.

## Materials and Methods

### Study Design and Participants

**Figure 1:**
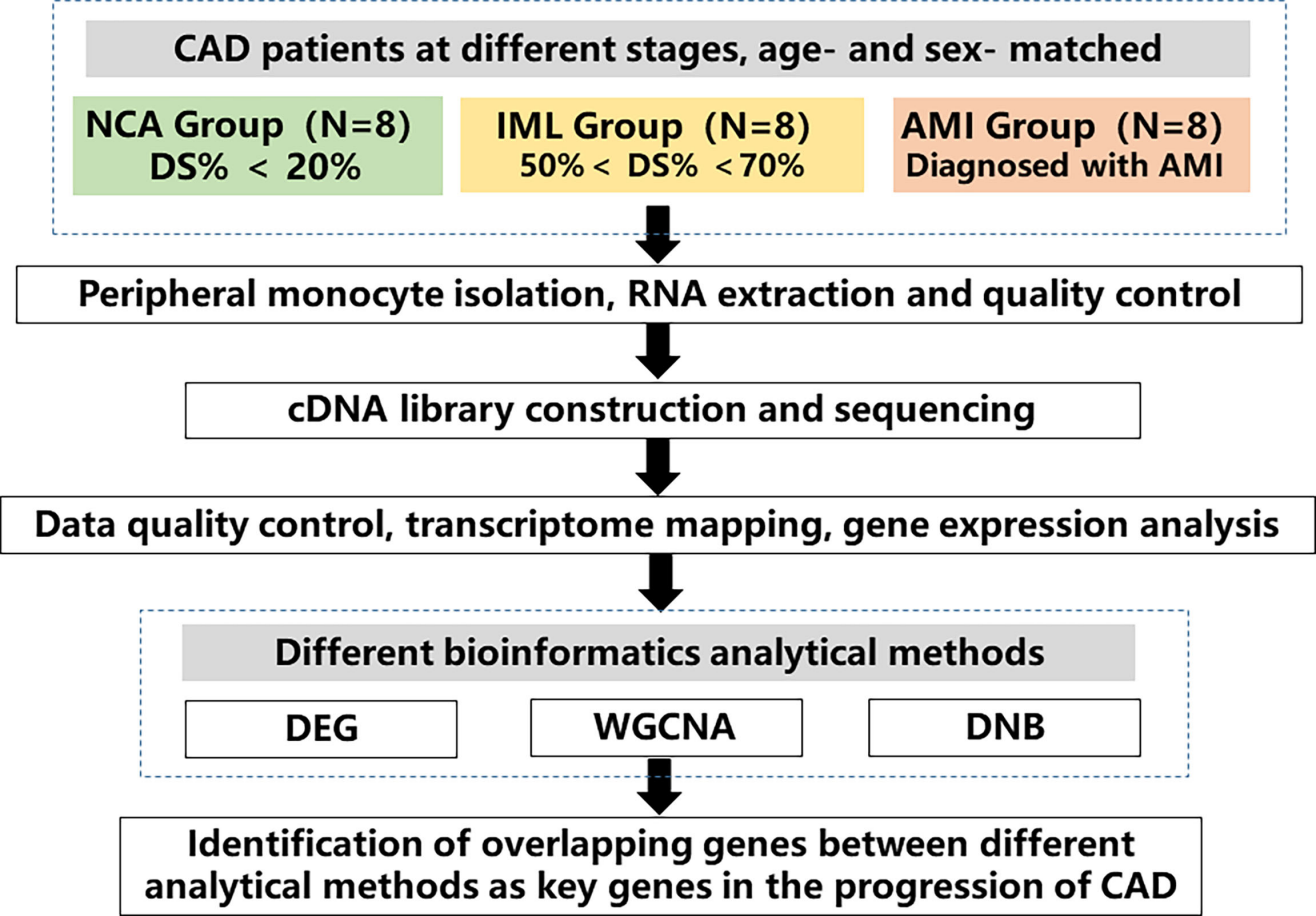
Study flow chart. A total of 24 age– and sex–matched coronary artery disease (CAD) patients at different disease stages were enrolled, which included 8 patients with normal coronary angiography (NCA), 8 patients with intermediated coronary lesion (IML), and 8 patients with acute myocardial infarction (AMI). Peripheral monocytes were isolated and RNA was extracted. Transcriptomic sequencing was performed, and various analytical methods were used to analyze gene expression profile across different groups. The overlapping genes between different analytical methods were identified as key genes in the progression of CAD. CAD, coronary artery disease, NCA, normal coronary angiography, IML, intermediate coronary lesion, AMI, acute myocardial infarction, DEG, differential expressed genes, WGCNA, weighted gene correlation network analysis, DNB, dynamic network biomarker.

The study design is shown in **Figure 1**. We collected blood samples from patients who agreed to provide blood samples from June 2011 to March 2015 at Fuwai Hospital. Eligible patients had symptoms indicating CAD and were receiving elective coronary angiography. We excluded patients with rheumatic heart disease, organic heart diseases and cardiomyopathy, severe liver and renal dysfunction, severe infectious diseases, malignant tumors, immune system and connective tissue diseases, and metabolic diseases, including hyperthyroidism and Cushing syndrome. Fasting blood samples and demographic information were collected at Fuwai Hospital after obtaining informed consent. Specifically, blood sample of patients in the AMI group were collected when they were rehospitalized due to ischemia symptoms. Sampling was performed prior to angiography in the fasting status. We randomly selected 8 patients with stable coronary heart disease and angiography–confirmed intermediate lesion (IML) (visual stenosis 50%–70%), as well as 8 age– and sex–matched patients with normal coronary angiography (NCA) and patients with AMI. AMI was diagnosed according to the third universal diagnosis of acute myocardial infarction, with cardiac biomarker (primarily cTn) elevation above 99% upper reference limit with ischemia symptoms, new–onset ischemic ST–T segment change or left bundle branch block, and Q wave formation. Patients in the IML group had objective evidence of ischemia such as chest pain and other myocardial ischemia symptoms or positive exercise stress test results. In recent years, the coronary physiology evaluation method fractional flow reserve (FFR) is the “gold standard” for identifying lesions with physiological significance. Recent studies have also confirmed that a high proportion of intermediate lesions, despite diameter stenosis <80%, may cause myocardial ischemia as detected by FFR. In addition, patients with intermediate lesions varied significantly in their long–term prognosis despite a similar degree of lesion stenosis. Therefore, the current study enrolled patients with intermediate lesions to represent the disease stage of “stable coronary heart disease.” The study protocol complied with the Declaration of Helsinki and was approved by the ethics committee of Fuwai Hospital (No. 2012–431).

### Peripheral Blood Monocyte Isolation, RNA Isolation, and Sequencing

Fasting blood samples were collected in the morning before the angiography procedure. Leukocytes were isolated by centrifugation, and monocytes were isolated by using EasySep™ Human CD14 Positive Selection Kit (#18058, Stemcell, USA) following the manufacturer’s instructions. RNA was extracted from leukocytes by using TRIzol^®^ Reagent (#15596018, Invitrogen, USA) and frozen at −80°C. The purity of RNA was assessed by NanoPhotometer Spectrophotometers (IMPLEN, USA), and the quantity and quality of RNA were assessed by Agilent 2100 Bioanalyzer and Agilent RNA 6000 Nano Assay Kits.

A total of 3 µg of qualified RNA was used to construct the cDNA library. mRNA was purified from total RNA using poly–T oligo–attached magnetic beads and fragmented with NEBNext First Strand Synthesis Reaction Buffer (5×). The sequencing was performed by Annoroad Gene Technology Corporation (Beijing) by using the Illumina Novaseq S2 platform (Illumina, USA) with the PE–150 module.

### Data Processing

Basic data processing included three major steps: data quality control, transcriptome mapping, and gene expression analysis. Raw data were filtered by the Cutadapt software to remove low–quality sequencing reads and generate high–quality data (clean reads). The human reference database and annotation files were downloaded from the ENSEMBL database (version Homo_sapiens.GRCh38.91.chr). The clean reads were then aligned to the reference genome by using HISAT2 v2.1.0 software.

Fragments per kilobase per million mapped fragments (FPKM) were calculated to assess gene expression level and used for subsequent further analysis. The algorithm for calculating FPKM is as follows:


$$FPKM=\frac{10^{3}*F}{NL/10^{6}}$$

where *F* is the number of reads mapped to the gene, *N* is the total number of mapped reads or fragments, and *L* is the gene length.

### Differential Expression Analysis and GO Enrichment Analysis

Differentially expressed gene (DEG) analysis was performed by using DESeq2 R packages, and genes with fold change ≥1.5 or fold change ≤0.67 and an adjusted *p*–value <0.05 were identified as differentially expressed genes. The volcano plot was used to visualize differentially expressed genes between groups and was generated by the ggplot packages. GO enrichment analysis was used to investigate the biological function of differentially expressed genes, which was performed and visualized on the Metascape website (https://metascape.org/gp/index.html#/main/step1) (27). The parameters were set as follows: min overlap of 3, *p*–value cutoff of 0.01, and minimum enrichment factor of 1.5. The top 20 enriched pathways were selected for visualization.

### WGCNA Analysis

The WGCNA analysis was performed by using the R package “WGCNA” (28), which mainly included three steps: construction of a gene coexpression network, identify gene modules, and gene module–clinical trait correlation analysis. The expression matrix was constructed with the original FPKM. We included genes with FPKM greater than zero in more than 8 samples. The soft–threshold power was chosen by the pickSoftThreshold function. The gene network was constructed by the blockwiseModules function, and the parameters were set as follows: minModuleSize = 30, reassignThreshold = 0, and mergeCutHeight = 0.25. The corresponding eigengenes were obtained to summarize the expression profile of each gene module. The association between each gene module and clinical trait was performed by using the Pearson’s correlation method, which correlate the module eigengenes with each clinical trait. Genes with absolute gene modulemembership > 0.8 and genetraitsignificance > 0.2 were identified as hub genes (29).

### DNB Analysis

**Figure 2:**
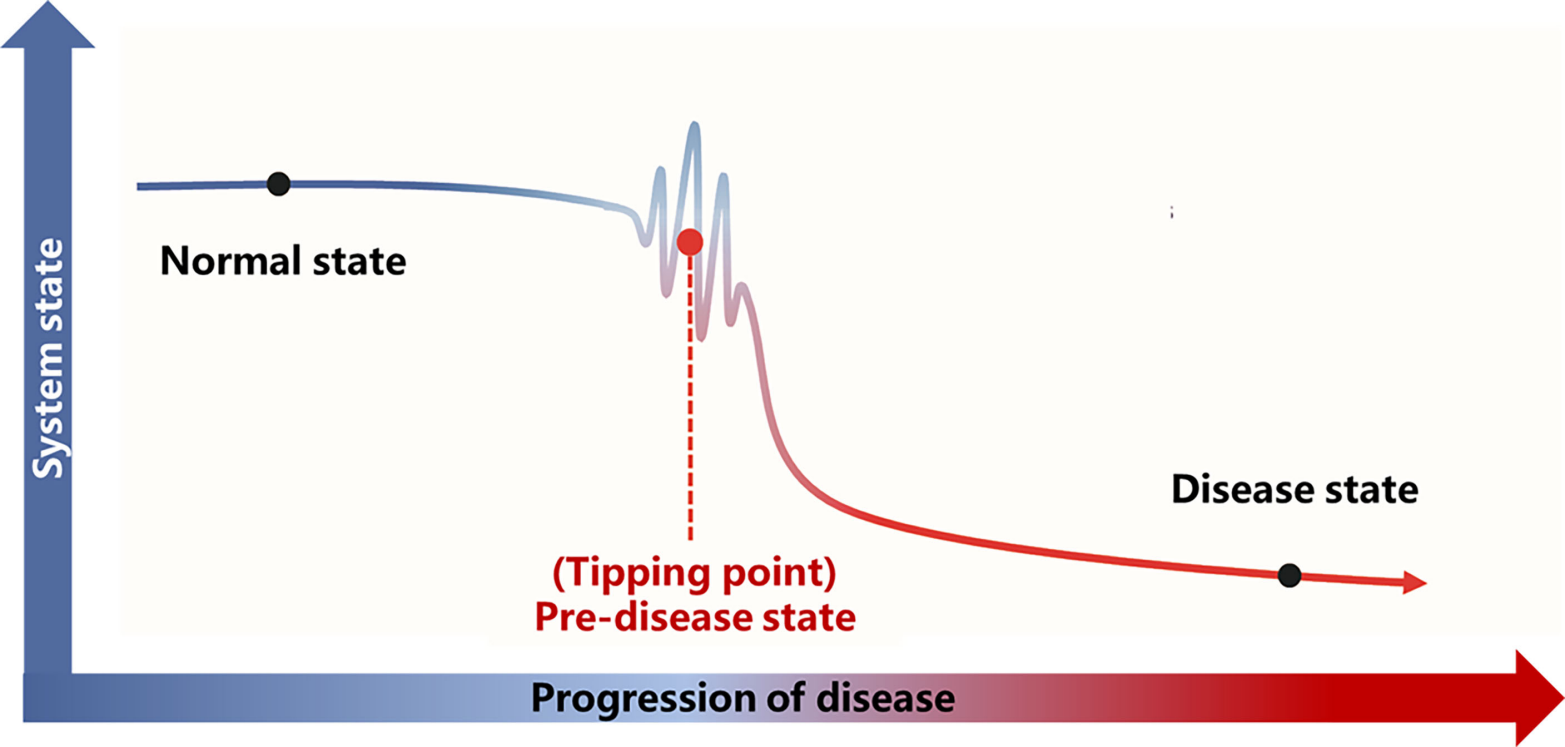
Illustration of DNB theory. Based on the DNB theory, there is a critical transition point (tipping point) during the progression of the disease. The disease progression process can be divided into three stages according to the tipping point: the normal state, the predisease state, and the disease state. The abbreviations are the same as above.

A detailed theoretical foundation and computational algorithm for DNB analysis were described previously (23). Based on the DNB theory, the progression of the disease is not smooth but abrupt (**Figure 2**), and there is a critical transition point after which the system shifts abruptly from one state to another. According to this transition point, the disease progression process can be divided into three stages: “normal state,” which is a relatively stable state where the disease undergoes gradual and slow change, “predisease state,” which is the limit of the normal state just before the transition to the disease state, and the “disease state,” which is another relatively stable state and is usually irreversible to the normal state. Based on the DNB theory, there exists a group of molecules (genes, proteins or metabolites, etc.) in the predisease state, which can be used for predicting disease. This group of molecules characterizes the dynamic features of the underlying system and are termed as DNB and satisfies the following three criteria:

1. The average standard deviations (SDs) of DNB molecules increase drastically.
2. The average Pearson’s correlation coefficients (PCCs) of DNB molecules drastically increase in the predisease state.
3. The average PCCs of molecules between DNB molecules and non–DNB molecules drastically decrease.

The above three criteria can which can be represented as a composite index (CI):


$$CI=\frac{SD_{d}\text{x}PCC_{d}}{PCC_{O}}$$

whereas SD*_d_* is the average standard deviation (SD) for molecules inside the DNB module, PCC*_d_* is the average Pearson’s correlation coefficient (PCC) in absolute value for molecules inside the module, and PCC*_o_* is the average PCC in absolute value for molecules between DNB and non–DNB. The CI is expected to increase abruptly and significantly before the critical transition to the disease state and can serve as an early warning signal.

### Statistical Analysis

SPSS 26.0 was used for statistical analysis. Continuous variables were expressed as mean ± standard deviation, and comparisons between groups were performed by analysis of variance. Categorical variables were expressed as frequency (percentage) using the chi–square test or Fisher’s exact test for comparison between groups. The difference was considered statistically significant when the bilateral P value was less than 0.05.

## Results

### Clinical Characteristics of the Recruited Patients

The baseline characteristics of the 8 IML patients and 8 gender– and–age–matched NCA and AMI patients are demonstrated in **Table 1**. Among the 24 patients, 17 (70.83%) were men, with a median age of 64 years. Additionally, no significant difference was found in terms of age, gender, and body mass index (BMI) between the different groups. As expected, CAD patients had a significantly higher rate of family history of CAD and hyperlipidemia, and more tended to be current smokers, but the difference was not statistically significant.

**Table 1:** Baseline characteristics across groups.

	NCA group (*N* = 8)	IML group (*N* = 8)	AMI group (*N* = 8)	*p*–value
Age (years)	59.75 ± 8.71	63.38 ± 9.04	63.25 ± 8.29	0.741
Male (%)	5 (62.50)	6 (75.00)	6 (75.00)	1.000
BMI (kg/m^2^)	24.75 ± 2.13	22.72 ± 2.28	26.00 ± 3.31	0.062
Diabetes (%)	2 (25.00)	1 (12.50)	3 (37.50)	0.837
Hypertension (%)	6 (75.00)	5 (62.50)	4 (50.00)	0.866
Dyslipidemia (%)	5 (62.50)	5 (62.50)	7 (87.50)	0.866
Family history of CAD (%)	0 (0.00)	7 (87.50)	1 (12.50)	0.557
Current smoker (%)	1 (12.50)	5 (62.50)	3 (37.50)	0.171
Alcohol (%)	4 (50.00)	1 (12.50)	2 (25.00)	0.402
LVEF (%)	64.95 ± 6.70	65.12 ± 4.36	58.43 ± 9.76	0.136

NCA, normal coronary angiography, IML, intermediate lesion, AMI, acute myocardial infarction, BMI, body mass index, CAD, coronary artery disease, LVEF, left ventricular ejection fraction.

### Differential Gene Expression Analysis of Different Phases of CAD Patients

**Figure 3:**
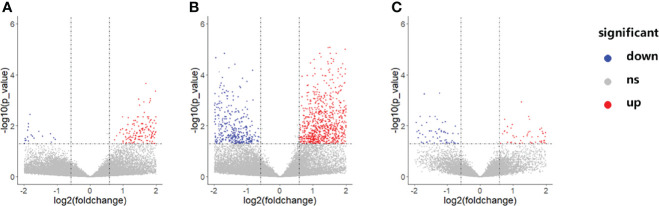
Volcano plot of differentially expressed genes between the IML and NCA groups **(A)**, AMI and NCA groups **(B)**, as well as AMI and IML group **(C)**. The abbreviations are the same as above.

Gene expression profiles differed significantly across groups. As shown in **Figure 3**, there were a total of 192 DEGs between the NCA and IML groups, 2,269 DEGs between the AMI and NCA groups, and 385 DEGs between the AMI and IML groups. To analyze the potential biological function of DEGs, the online analytic tool Metascape was used, and differential genes were uploaded on the website. The top enriched pathways of the DEGs between the IML group and the NCA group were associated with cell adhesion, cell migration, and cell response to inflammation, the top enriched pathways of the DEGs between the AML group and the NCA group were associated with cellular response to stress and cellular cytokine production, and the top enriched pathways of the DEGs between the AML group and the IML group were associated with mRNA metabolic process, cellular signal transduction, and cellular response to growth factor stimulus (**Supplementary Figures S1–S3**).

### WGCNA Analysis

**Figure 4:**
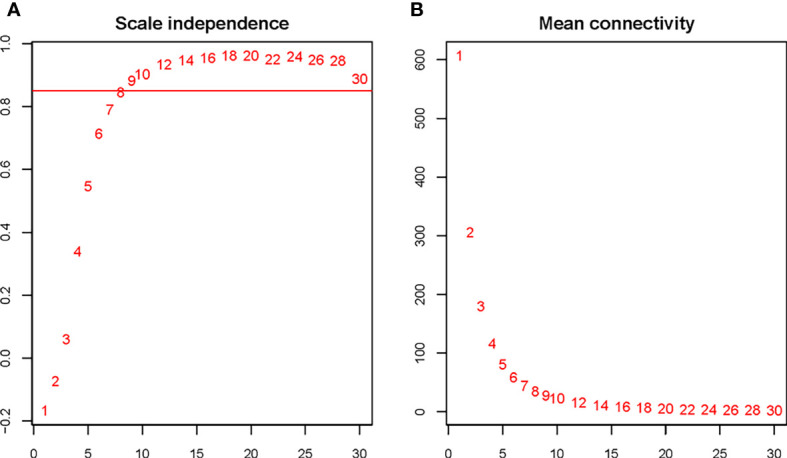
Determination of the soft threshold for the WGCNA analysis. Analysis of the scale–free index for various soft–threshold powers **(A)** and mean connectivity **(B)** for various soft–threshold powers. The abbreviations are the same as above.

**Figure 5:**
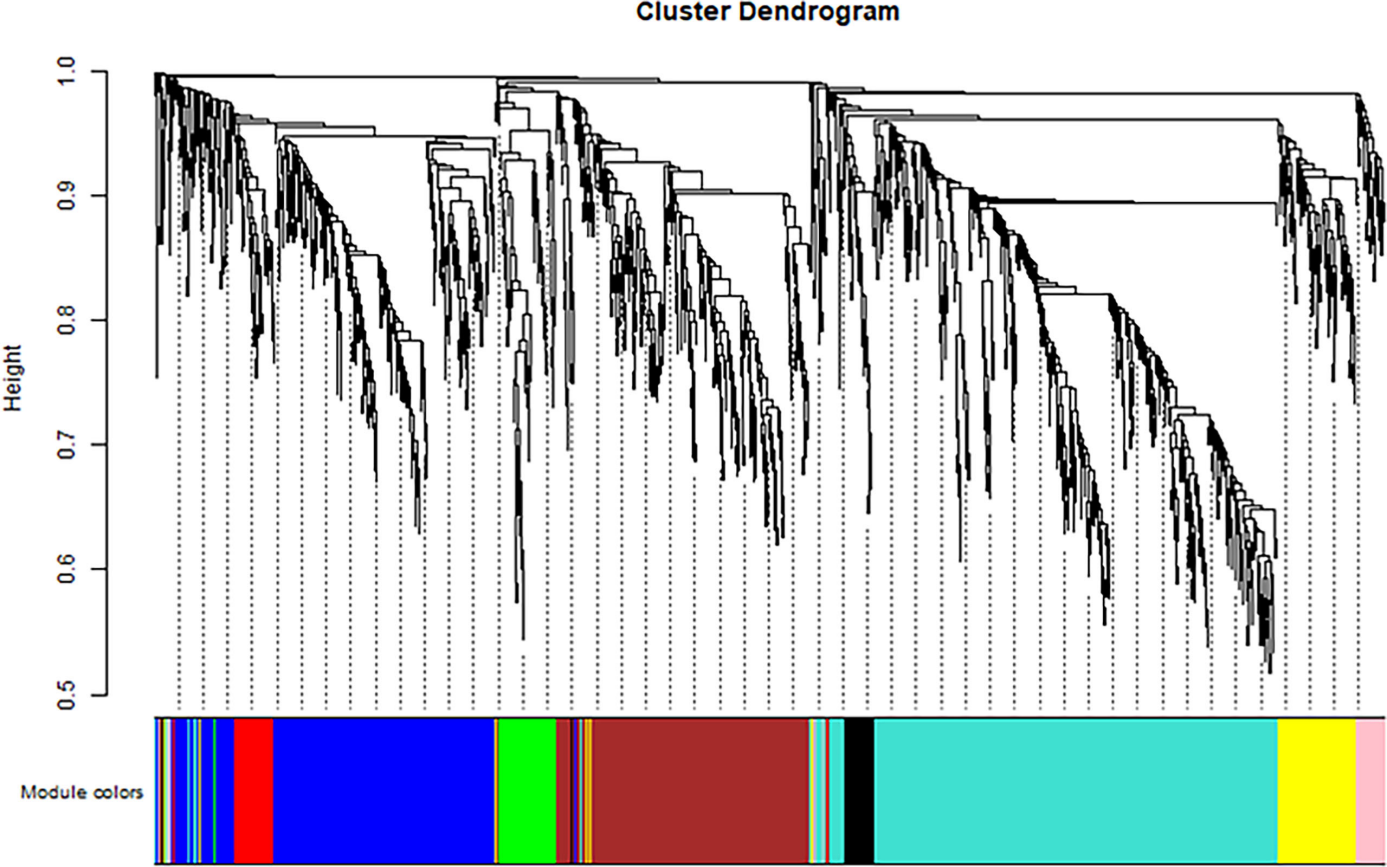
Clustering dendrogram of all differentially expressed genes based on dissimilarity, with an assigned color for each gene module.

The soft threshold of 8 was used to construct the scale–free network (**Figure 4**). Nine modules were identified based on average hierarchical clustering and dynamic tree clipping (**Figure 5**). The number of genes in each gene module is shown in **Table 2**.

**Table 2:** The number of genes and median MM and GS in each gene module.

Gene module	No. of genes	MM	GS
Gray	12	0.540	0.296
Turquoise	575	0.812	0.441
Blue	367	0.761	0.380
Brown	330	0.803	0.681
Yellow	118	0.854	0.613
Green	77	0.844	0.599
Red	57	0.832	0.430
Black	50	0.829	0.290
Pink	40	0.796	0.508

MM, module membership; GS, gene significance.

**Figure 6:**
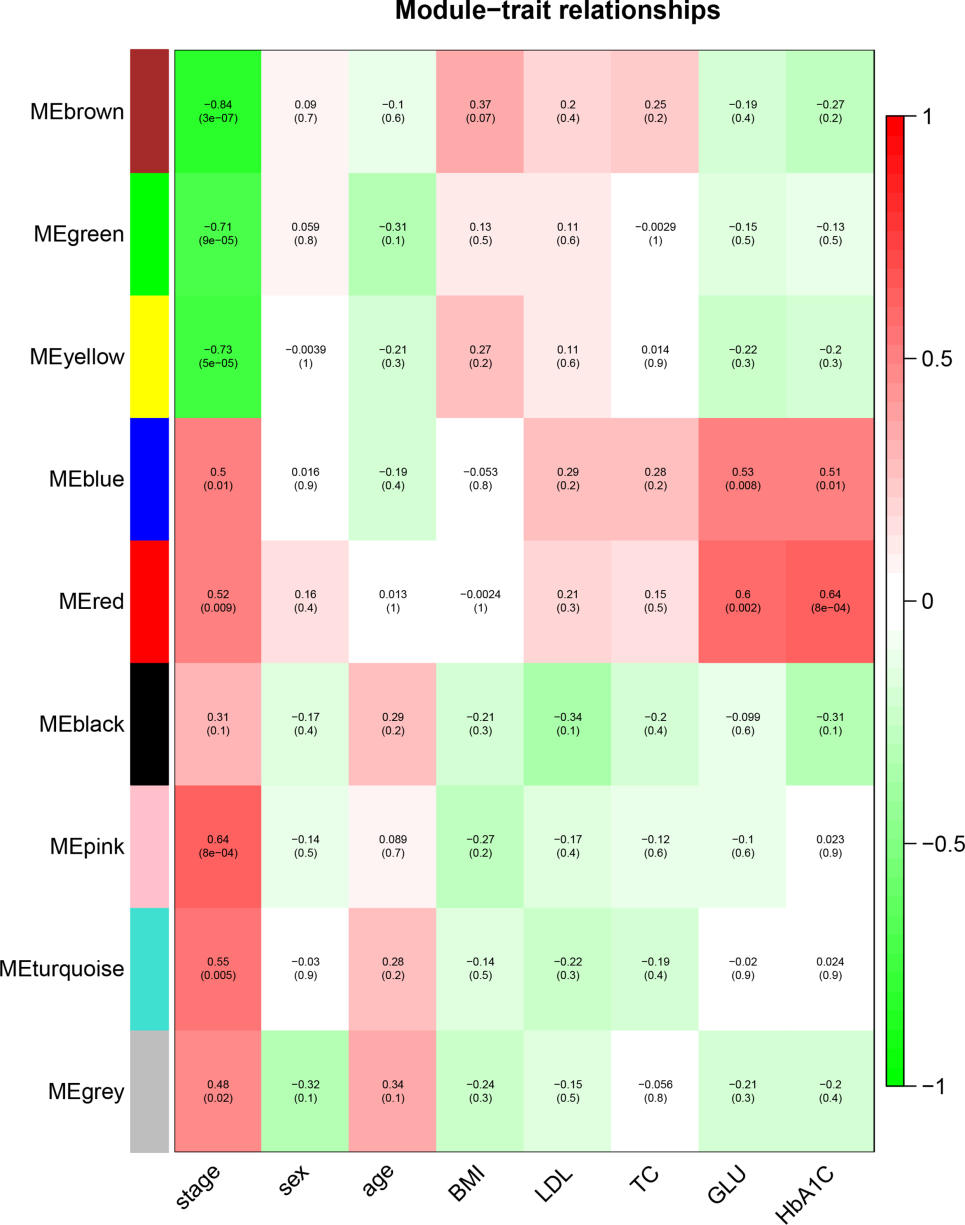
Identification of the gene module associated with clinical traits. Heatmap of the correlation between the module eigengenes and clinical traits of CAD patients. Disease stage is a trichotomous variable (NCA, IML, and AMI). The number in each cell is the correlation coefficient (the corresponding *p*–value). The abbreviations are the same as above.

To identify the genes associated with the progression of atherosclerosis, we evaluated the association between each gene module and clinical trait by calculating the module significance (MS) for each module–trait correlation (**Figure 6**). Disease stage is a trichotomous variable that indicates NCA, IML, and AMI. The pink module had the highest positive association with disease stage (*r* ^2^ = 0.64, *p* = 8e−04), followed by the turquoise module (*r* ^2^ = 0.55, *p* = 0.005).

Genes within each gene module, as well as the corresponding module membership and gene significance, are shown in **Supplementary Table S1**. The top 3 GO–BP–enriched pathways in pink module are endoplasmic reticulum organization, regulation of alpha–beta T cell, and peptidyl–serine phosphorylation (Figure not shown). The top enriched pathways in the turquoise module are associated with cell response to inflammatory stimuli, lymphocyte activation, cytokine production, and the apoptotic signaling pathway (**Supplementary Figure S4**), which has been reported to be closely associated with the atherosclerotic process.

The scatter plot for gene significance and module membership for the above gene modules are shown in **Supplementary Figure S5**.

### DNB Genes

**Figure 7:**
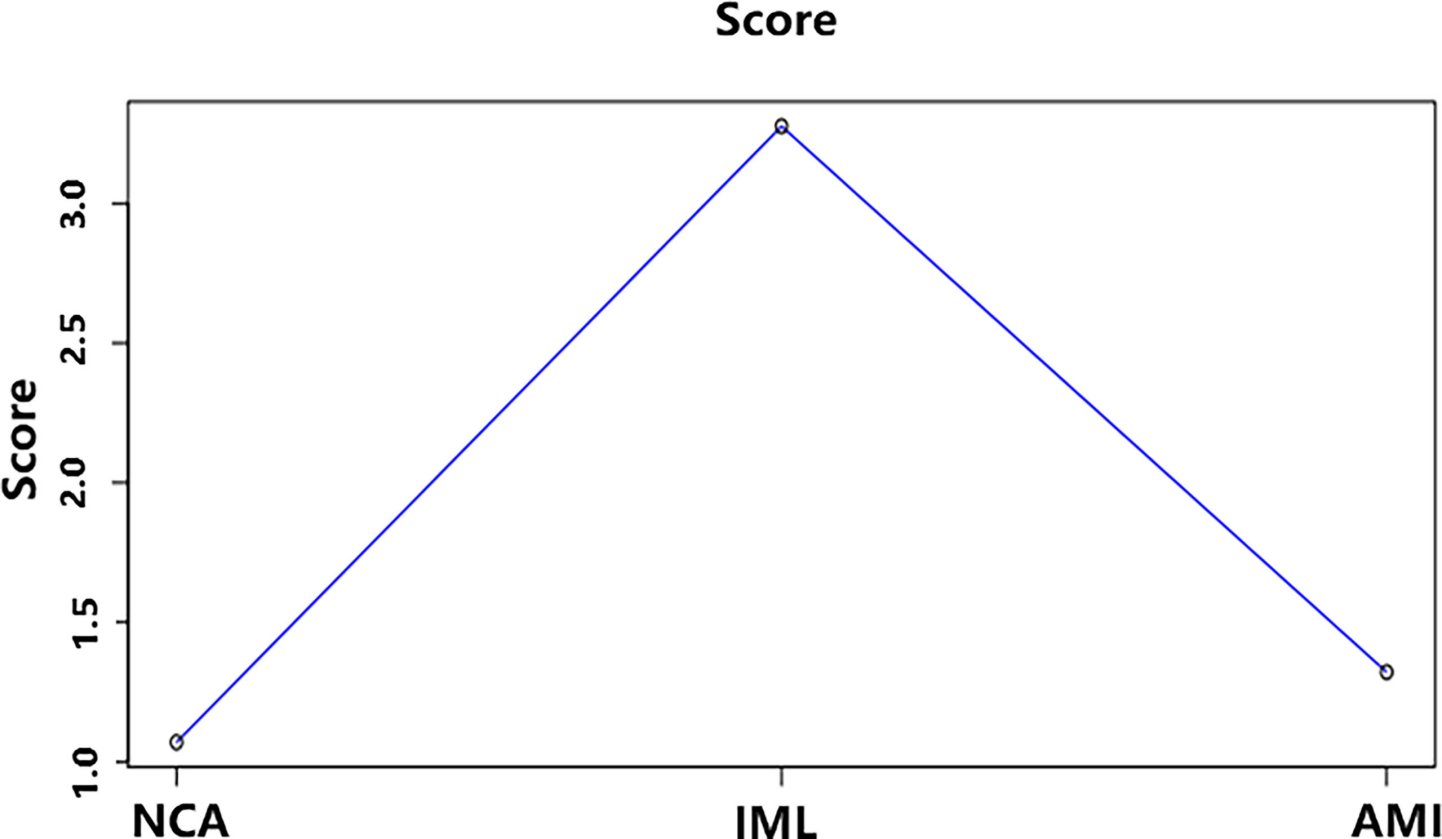
The average DNB score and corresponding parameters for the three groups. *x*–axis represents the three groups, and *y*–axis represents the average DNB score for each group.

The average DNB score for each group is shown in **Figure 7**. The average DNB score of the IML group is greater than that of the NCA and AMI groups, which indicates that the critical transition point of atherosclerosis is at the IML stage. This is also consistent with the pathophysiological process of atherosclerosis progression in clinical practice.

There are a total of 103 DNB genes (**Supplementary Table S2**). The top 3 GO–BP enrichment pathways included regulation of activation of Janus kinase activity, regulation of cell adhesion, and negative regulation of proteolysis (**Supplementary Figure S6**).

### Genes Potentially Associated with CAD progression

**Figure 8:**
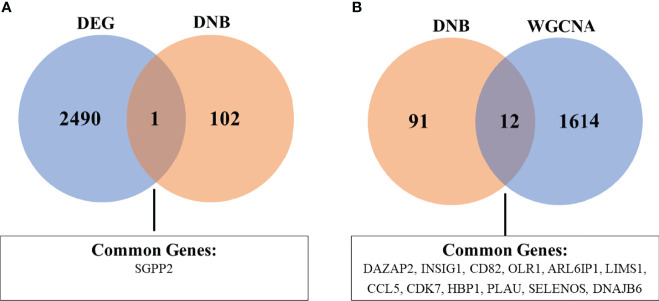
Venn diagram showing the common genes between DEG and DNB **(A)**, as well as DNB and WGCNA analyses **(B)**. The abbreviations are the same as above.

To identify robust biomarkers that may serve as early warning signals of AMI, we analyzed the common genes (overlapping genes) between differentially expressed gene sets and DNB gene sets and identified the SGPP2 genes that may play key roles in the process of atherosclerosis progression (**Figure 8**).

**Figure 9:**
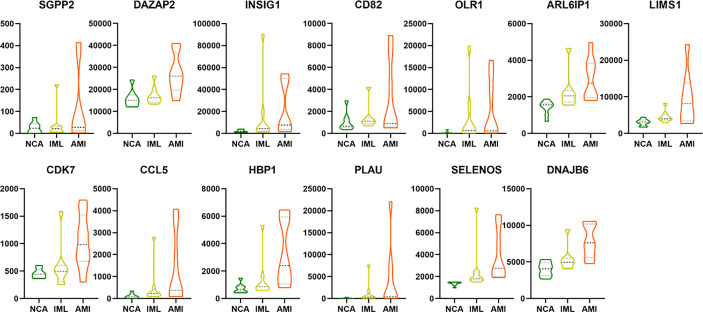
The violin plot showing the gene expression level of the 13 overlapping genes between DEG/WGCNA and DNB analyses. The abbreviation.

We also analyzed the overlapping genes between DNB gene sets and WGCNA gene sets and identified the following 12 genes: DAZAP2, INSIG1, CD82, OLR1, ARL6IP1, LIMS1, CCL5, CDK7, HBP1, PLAU, SELENOS, and DNAJB6. Gene expression levels are expressed in the violin plot in **Figure 9**.

## Discussion

CAD remains the leading cause of disease burden worldwide. Elevated LDL–c levels are the well–established principal risk factor for the onset and progression of CAD. However, a substantial number of patients still suffer from adverse cardiovascular events despite the reduction of LDL–c level to 40 mg/dl or less (30, 31), suggesting additional “residual cardiovascular risks” are yet to be explored.

In the microenvironment of coronary atherosclerotic plaques, the main type of immune cell is the macrophage, which is differentiated from peripheral blood monocyte. In atherogenic conditions, circulation monocytes sense changes in the microenvironment and adopt specific gene expression in response. These monocytes are then recruited to the subintimal layers of the artery wall and differentiated into macrophages. Therefore, studying the gene expression of peripheral blood monocytes in different stages of CAD through RNA–sequencing will help to reveal the underlying mechanism.

Disease progression is a complex nonlinear process, which is not necessarily “smooth” but “abrupt” (23). There is usually a drastic change during disease progression, which causes the critical transition from the normal state to the disease state (**Figure 2**).

Traditional bioinformatics methods primarily compare the molecular characteristics between disease and normal state and poorly detect the predisease state, or accurately predict the onset/deterioration of disease before its occurrence, due to the similarity of molecular characteristics between the pre–disease state and the normal state. To overcome this limitation, a novel computational algorithm, dynamic network biomarkers, was proposed to detect the critical transition point in the complex biological process, such as the progression of atherosclerosis in our study. The DNB theory was based on solid nonlinear dynamic system theory and was reported to serve as an early–warning signal prior to disease deterioration.

Several of these genes have been reported to be closely associated with the onset or progression of atherosclerosis, further demonstrating the robustness of our conclusion.

C–C motif chemokine ligand 5 (CCL5) encodes a 68–amino acid chemokine, which functions as a chemoattractant for blood immune cells and the natural ligand for the chemokine receptor chemokine (C–C motif) receptor 5 (CCR5). CCL5 is involved in a wide range of inflammatory processes, including advanced atherosclerosis and myocardial reperfusion injury. CCL5 has been detected in atherosclerosis plaque (32). CCR5 deficiency reduces the development of diet–induced atherosclerosis in mice (33). Inhibition of CCL5 reduces myocardial reperfusion in atherosclerosis mice (34).

Oxidized low–density lipoprotein receptor 1 (OLR1) encodes a low–density lipoprotein receptor, which binds, internalizes, and degrades oxidized low–density lipoprotein. The previous meta–analysis demonstrated a significant association between OLR1 gene polymorphisms and CAD risk (35). OLR1 promotes endothelial dysfunction by inducing pro–atherogenic signaling *via* the endothelial uptake of oxidized LDL (oxLDL), which contributes to the initiation, progression, and destabilization of atheromatous plaques (36). In addition to its expression in endothelial cells, OLR1 is also expressed in immune cells, including macrophages, lymphocytes, and neutrophils, further implicating this receptor in multiple aspects of atherosclerotic plaque formation. In conclusion, OLR1 holds promise as a novel diagnostic and therapeutic target for atherosclerosis and CHD.

Plasminogen activator, urokinase (PLAU) encodes a secreted serine protease that converts plasminogen to plasmin. PLAU has been implicated in a broad spectrum of biological and pathological processes, including chemotaxis, cell adhesion, migration and growth, fibrinolysis, proteolysis, angiogenesis, inflammation, and neointima formation (37). PLAU has also been reported to be associated with atherosclerosis plaque formation and AMI: macrophage–specific overexpression of the PLAU gene accelerated atherosclerosis, coronary artery occlusions, and premature death in ApoE^−/−^ mice (38). PLAU has already been reported to be closely associated with AMI: an SNP rs4065 of the PLAU gene is associated with AMI risk in the Chinese Han population (39). Taken together, the above evidence suggests the PLAU gene as a novel therapeutic target for the treatment of atherosclerosis.

Several genes have been reported to be involved in metabolic or inflammatory processes and, therefore, may potentially be involved in the pathophysiological process of atherosclerosis. However, this hypothesis still needs to be demonstrated in future studies.

Sphingosine–1–phosphate phosphatase 2 (SGPP2) is differentially expressed between both the AMI and NCA groups, as well as the AMI and IML groups. SGPP2 encodes sphingosine–1–phosphate phosphatase 2, which can degrade sphingosine 1–phosphate (S1P) to produce sphingosine. SGPP2 is expressed in human umbilical vein endothelial cells and neutrophils (40). The results of previous studies suggest that the *SGPP2* gene may be involved in the onset and progress of atherosclerosis. The *SGPP2* gene affects the endothelial barrier function *via* altering the expression of interleukin 1–β (IL–1β) (40) in endothelial cells. IL–1β is a proinflammatory cytokine that can induce endothelial cell inflammation and destroy the endothelial barrier function (41). In addition, SGPP2 is involved in the inflammatory response process: SGPP2 knockout mice showed reduced expression of proinflammatory factors, including IL–6, TNF–α, and IL–1β, molecular mechanisms, and inhibition of inflammation–induced signal transducer and activator of transcription 3 (STAT–3) signal pathway activation related. The activation of the STAT–3 signaling pathway plays an important role in the process of macrophage inflammation and polarization (42). Endothelial barrier dysfunction, inflammatory cell infiltration, and release of inflammatory factors are the key factors leading to plaque progression and rupture.

Insulin–induced gene 1 (INSIG1) gene encodes an endoplasmic reticulum membrane protein that regulates cholesterol metabolism, lipogenesis, and glucose homeostasis in various tissues (43). INSIG1 acts as a negative regulator of cholesterol biosynthesis by mediating the retention of the SCAP–SREBP complex in the endoplasmic reticulum, thereby blocking the processing of sterol regulatory element–binding proteins (SREBPs) (44). INSIG1 gene single nucleotide polymorphisms were associated with coronary heart disease risk in the Chinese Han population (45). Knockdown of INSIG1 resulted in a significant reduction of cholesterol efflux to HDL (46). INSIG1 variation may contribute to statin–induced changes in plasma TG in a sex–specific manner (47).

CD82 encodes a membrane glycoprotein, a member of the transmembrane 4 superfamily. The primary research area of CD82 is tumors, and CD82 has been recognized as a tumor metastasis suppressor gene (48). CD82 inhibits pathological angiogenesis. Endothelial cells CD82 knockout enhanced the migration and invasion capabilities of endothelial cells (49). CD82 also plays a key role in the regulation of endothelial–monocyte interactions, which include monocyte recruitment and migration (50). The above studies suggest that CD82 may affect atherosclerotic plaque formation, which requires validation in future studies.

Selenoprotein S (SELENOS) encodes a transmembrane protein, which is involved in the degradation process of misfolded proteins in the ER and may also have a role in inflammation control. SELENOS has been reported to be associated with the risk of diabetes: genetic polymorphisms of SELENOS genes are associated with diabetes risk in the Chinese population (51, 52). The serum source of SELENOS is primarily from hepatocytes, and the serum level of SELENOS was associated with the risk of DM and its macrovascular complications (53). Given that SELENOS is closely associated with inflammation, oxidative stress, as well as glucose metabolism (53), the above evidence indicates SELENOS played a key role in the pathophysiology process of AS, which requires future validation.

The biological function and their roles in atherosclerosis progression for the other genes, including DAZAP2, ARL6ip, CDK7, LIMS, HBP1, and DNAJB6, remain to be investigated. The current study has several limitations: Firstly, sample size of the current study was limited, and 8 samples were tested using RNA–seq for each group. Setting up biological replicates is necessary to eliminate errors in sequencing studies, and sequencing technique or statistical tools cannot fully eliminate biological variability. One recent study recommended no fewer than 6 biological replicates should be included in a single group in RNA–seq study (54). Also, to compensate for this deficiency, the DNB algorithm was applied. One advantage of the DNB algorithm is that it can identify the critical transition stage even based on small samples of high–throughput data (55). The first publication proposed and demonstrated the validity of the DNB algorithm by detecting the “early warning signals” prior to acute lung injury in carbonyl chloride inhalation–induced acute lung injury, based on 2–5 samples of lung tissue transcriptomic data at each sampling period (23). Secondly, since the current study was a retrospective study, future studies are required to validate whether the key genes in our study can predict adverse events in a prospective cohort, which is an ongoing project. Thirdly, since the current study was based on high–throughput sequencing data, quantitative validation of the gene expression level is required based on the qPCR method. Finally, basic studies are needed to explore the biological functions of the key genes identified in our study.

## Conclusion and Future Prospects

In conclusion, based on the peripheral blood mononuclear cell transcriptome sequencing data from patients at different disease progression stages of coronary artery disease, combined with traditional DEG analysis, WGCNA analysis, and novel DNB methods, the current study identified a total of 13 genes that may play key roles involved in the progression of atherosclerotic plaque and provides new insights for early warning biomarkers and potential underlying mechanisms underlying the progression of CAD.

## Data Availability Statement

The datasets presented in this study can be found in online repositories. The names of the repository/repositories and accession number(s) can be found below: https://www.ncbi.nlm.nih.gov/geo/, GSE166780.

## Ethics Statement

The studies involving human participants were reviewed and approved by the ethics committee of Fuwai Hospital. The patients/participants provided their written informed consent to participate in this study.

## Author Contributions

CS and ZQ carried out the sequence data analysis, participated in the sequence alignment, and drafted the manuscript. LC and JG created the method of the DNB analysis. SY, XB, CW, RZ, LJ, and QL participated in the design of the study and performed the statistical analysis. RF and KD conceived of the study and participated in its design and coordination and helped to draft the manuscript. All authors read and approved the final manuscript.

## Funding

This work was supported by the CAMS Innovation Fund for Medical Sciences (CIFMS) (Grant No. 2021–I2M–1–008) awarded to KD and was supported by The Natural Science Foundation of China (No. 81870277). This work was also supported by Special Fund for Science and Technology Innovation Strategy of Guangdong Province (Nos. 2021B0909050004, 2021B0909060002); Major Key Project of PCL (No.PCL2021A12); JST Moonshot R&D Project (No.JPMJMS2021).

## Conflict of Interest

The authors declare that the research was conducted in the absence of any commercial or financial relationships that could be construed as a potential conflict of interest.

## Publisher’s Note

All claims expressed in this article are solely those of the authors and do not necessarily represent those of their affiliated organizations, or those of the publisher, the editors and the reviewers. Any product that may be evaluated in this article, or claim that may be made by its manufacturer, is not guaranteed or endorsed by the publisher.
