# Supplementary figures and images for: Identification of Key Genes as Early Warning Signals of Acute Myocardial Infarction Based on Weighted Gene Correlation Network Analysis and Dynamic Network Biomarker Algorithm
Source: Front Immunol. 2022 Jun 20;13:879657. doi: 10.3389/fimmu.2022.879657 (PMC9251518; doi:10.3389/fimmu.2022.879657)

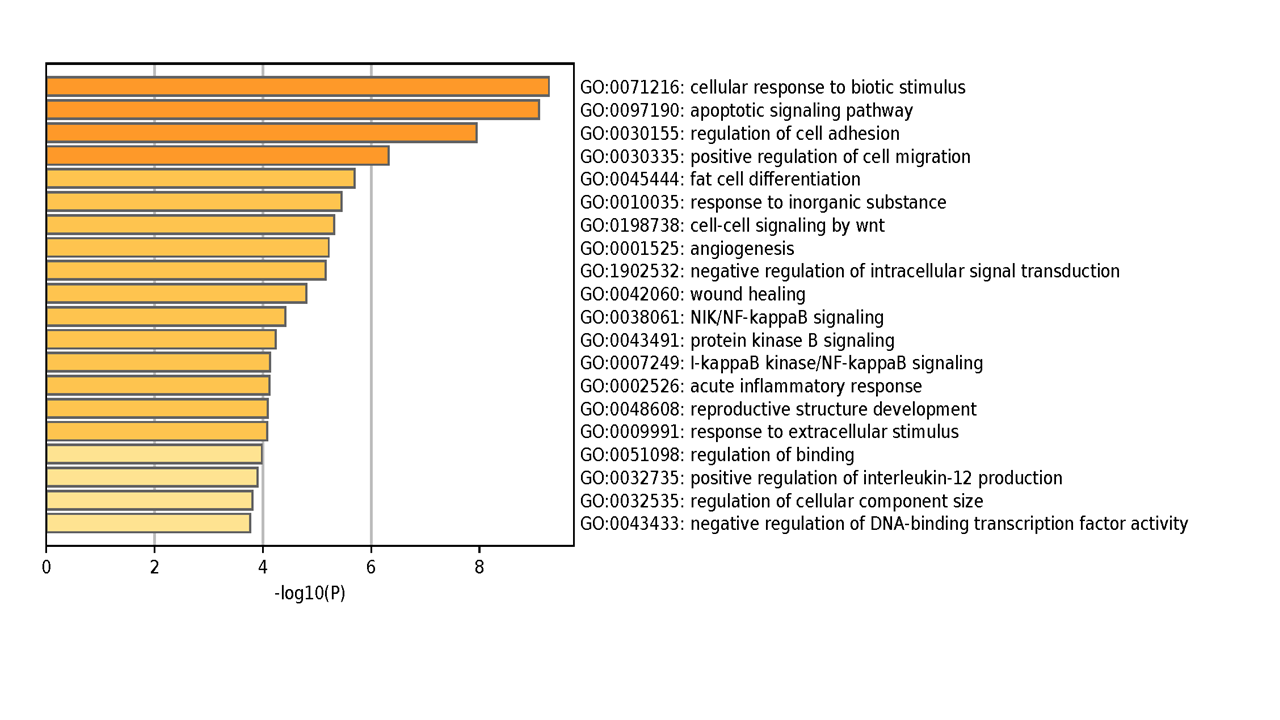

Supplement: Supplementary file 1 [file Image_1.tif]

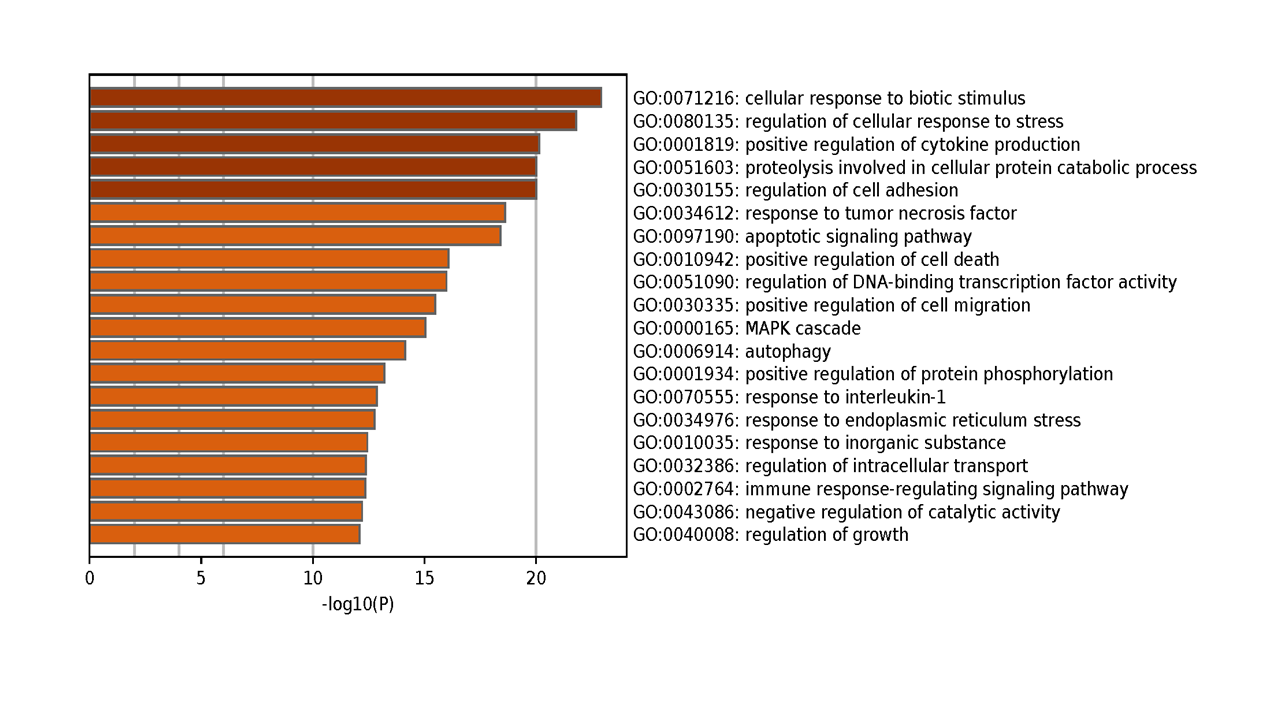

Supplement: Supplementary file 2 [file Image_2.tif]

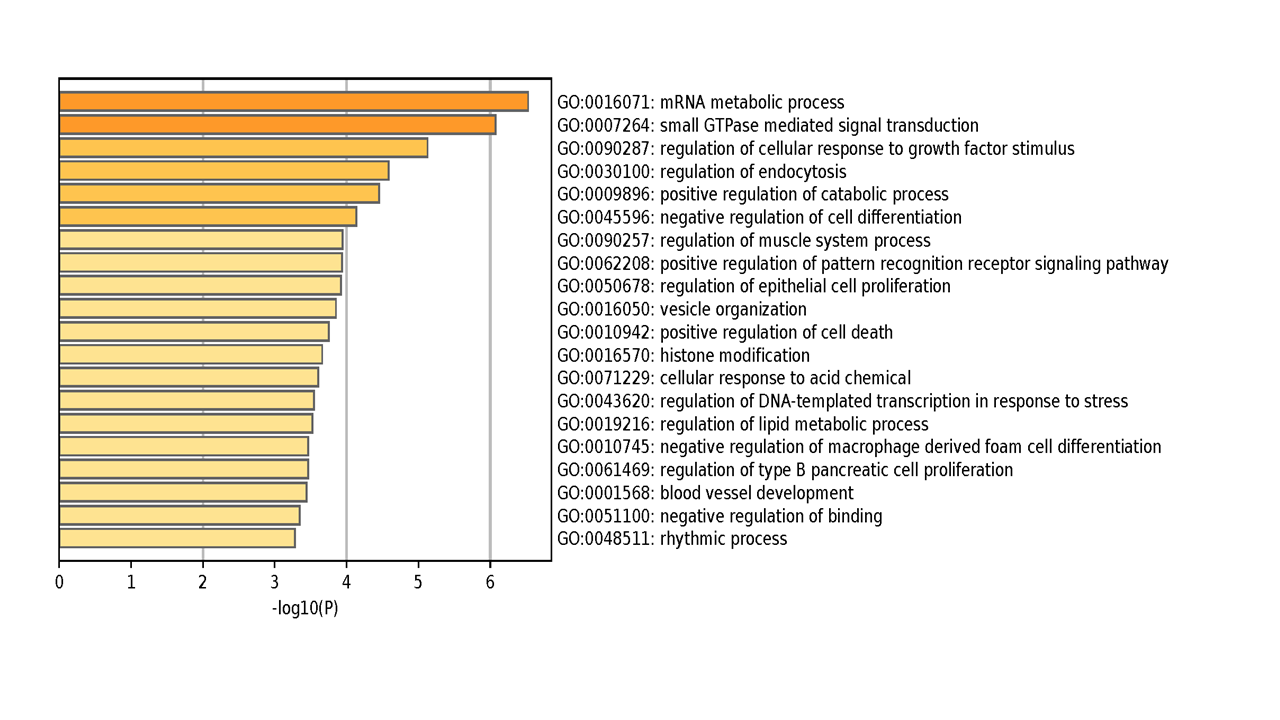

Supplement: Supplementary file 3 [file Image_3.tif]

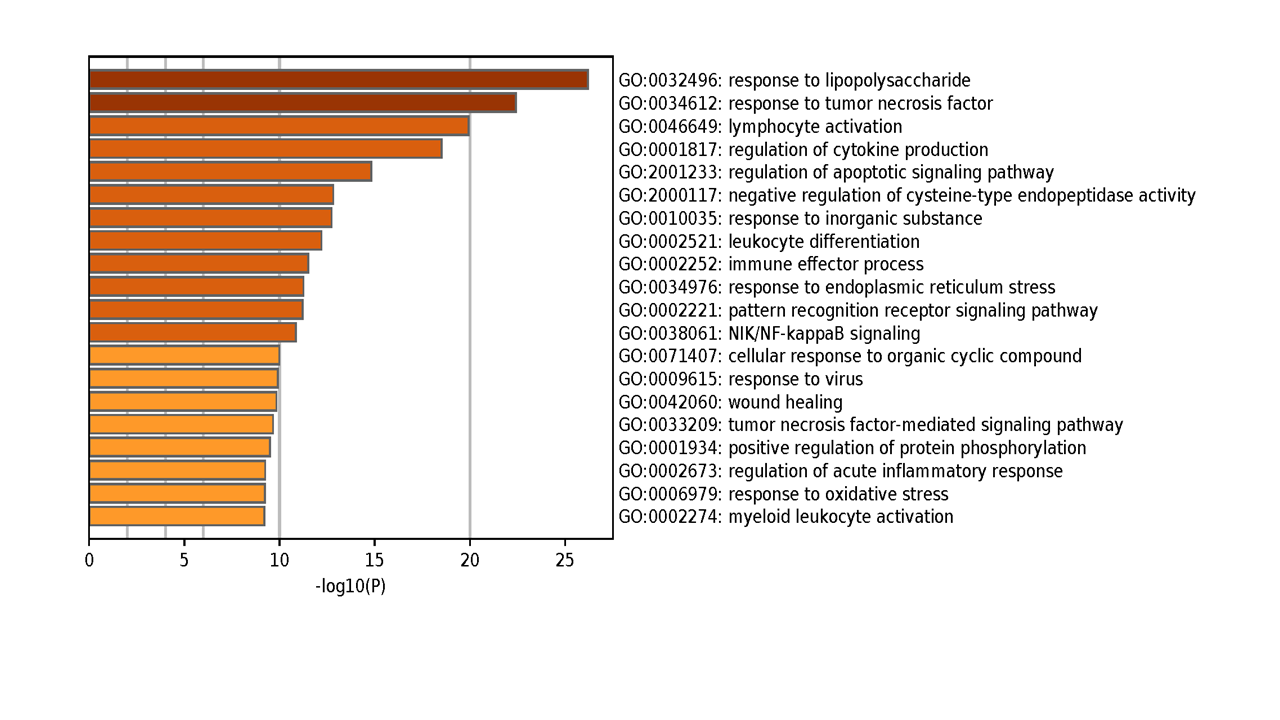

Supplement: Supplementary file 4 [file Image_4.tif]

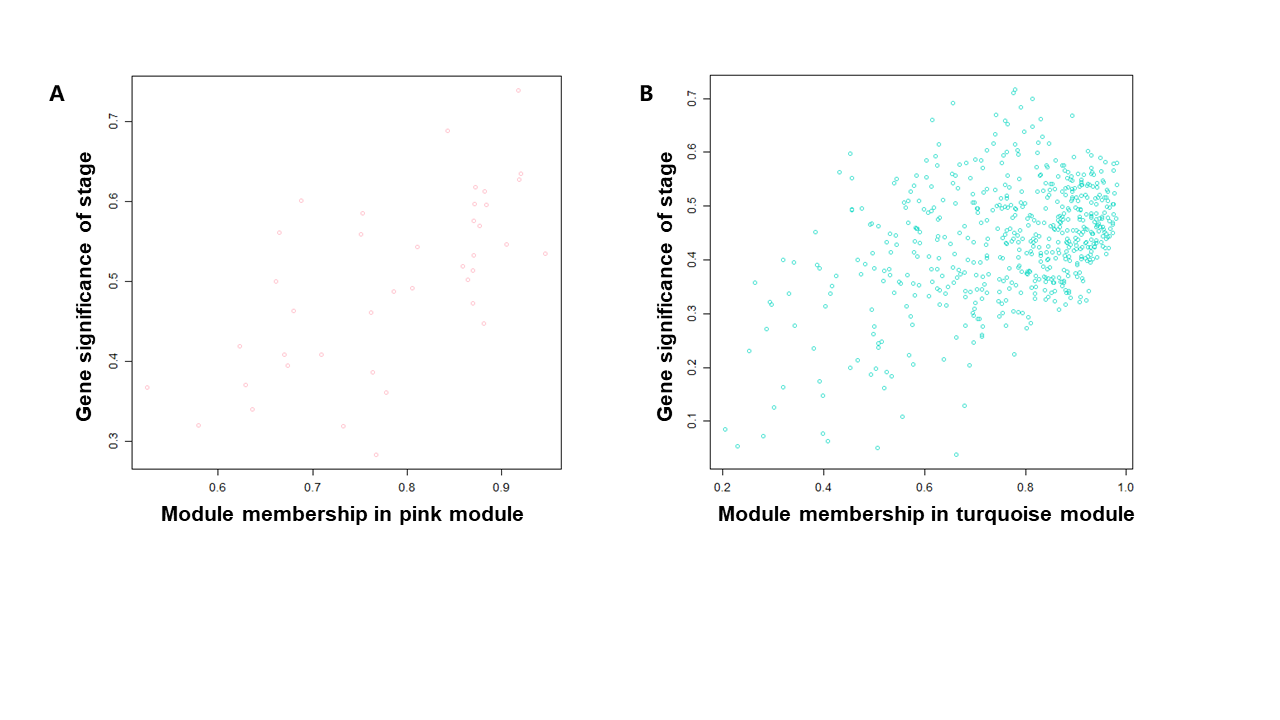

Supplement: Supplementary file 5 [file Image_5.tif]

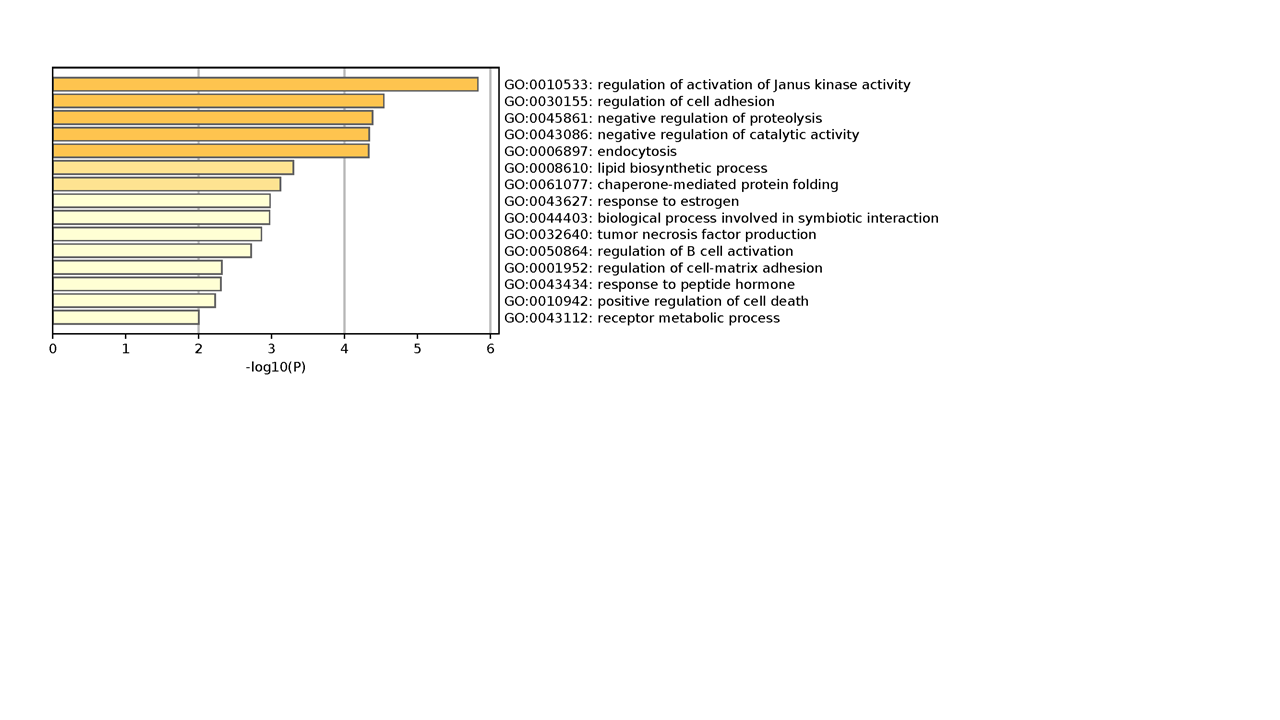

Supplement: Supplementary file 6 [file Image_6.tif]
